# Investigating the relationship of porcine reproductive and respiratory syndrome virus RNA detection between adult/sow farm and wean-to-market age categories

**DOI:** 10.1371/journal.pone.0253429

**Published:** 2021-07-02

**Authors:** Yiqun Jiang, Qing Li, Giovani Trevisan, Daniel C. L. Linhares, Cameron MacKenzie

**Affiliations:** 1 Department of Industrial and Manufacturing System Engineering, Iowa State University, Ames, IA, United States of America; 2 College of Veterinary Medicine, Iowa State University, Ames, IA, United States of America; University of Nicolaus Copernicus in Torun, POLAND

## Abstract

Porcine reproductive and respiratory syndrome (PRRS) is a disease caused by the PRRS virus (PRRSV) that has spread globally in the last 30 years and causes huge economic losses every year. This research aims to 1) investigate the relationship between the PRRSV detection in two age categories (wean-to-market and adult/sow farm), and 2) examine the extent to which the wean-to-market PRRSV positive rate forecasts the adult/sow farm PRRSV positive rate. The data we used are the PRRSV RNA detection results between 2007 and 2019 integrated by the US Swine Disease Reporting System project that represent 95% of all porcine submissions tested in the US National Animal Health Network. We first use statistical tools to investigate to what extent the increase in PRRSV positive submissions in the wean-to-market is related to the PRRSV increase in adult/sow farms. The statistical analysis confirms that an increase in the PRRSV positive rate of wean-to-market precedes the increase in the adult/sow farms to a large extent. Then we create the dynamic exponentially weighted moving average control charts to identify out-of-control points (i.e., signals) in the PRRSV rates for both wean-to-market and adult/sow farms. This control-chart-based analysis finds that 78% of PRRSV signals in the wean-to-market are followed by a PRRSV rate signal in the adult/sow farms within eight weeks. We expect that our findings will help the producers and veterinarians to justify and reinforce the implementation of bio-security and bio-contaminant practices to curb disease spread across farms.

## Introduction

Porcine reproductive and respiratory syndrome (PRRS) is a disease caused by the PRRS virus (PRRSV) [1]. The disease was first reported during the late 1980s concomitantly in the United States and Europe [2, 3]. Shortly after, the disease was successfully reproduced experimentally, and the etiologic agent was isolated [3–5]. In the last 30 years, PRRSV has spread globally [1, 6–12], and only a few countries are still PRRS-free. Attributed economic losses to PRRSV are €3–€160 per sow in Europe and $664 million yearly for the U.S. swine herd [13].

In 2018, a project, named as Swine Disease Reporting System (SDRS), was established in the United States to report PRRSV RNA detection results by a reverse-transcription polymerase chain reaction (RT-PCR) test on porcine samples submitted for testing at four major swine-centric veterinary diagnostic laboratories that represent 95% of all porcine submissions tested in the US National Animal Health Network [12]. The SDRS successfully integrated more than ten years of historical data, from 2007 to the present, and proactively incorporated newly generated data by participant veterinary diagnostic laboratories (VDLs). The percentage of positive submissions for PRRSV RNA detected by RT-PCR followed a cyclic pattern of detection with a higher percentage of positive submissions occurring during colder months and the fewer during warmer months [14]. The percentage of positive results could be statistically modeled to forecast the upcoming year’s weekly expected percentage of positive submissions and to monitor changes from expected baselines [14]. During 2018, PRRSV detection was more than expected, and data from different age categories were used to investigate the source for this increased detection further. A repetitive pattern over time showed that an increase in the percentage of positive results for the age category adult/sow farm during the second half of the year was preceded by an increase in the percentage of positive submissions for the age category wean-to-market [14].

The age category adult/sow farm reports RT-PCR PRRSV RNA detection from samples identified from sites that house replacement animal, board stud, breeding herd, and suckling piglets, or both; and the age category wean-to-market reports results for sites that house animals in the growing phase, i.e., from weaning to market [12]. One would expect that a movement of piglets from the age category adult/sow farm to the age category wean-to-market might lead to an increase in the detection of PRRSV in the latter category. The opposite was unexpectedly observed in the SDRS data and raised the concern that wean-to-market animals play a major role in the dynamics of PRRSV detection during the second half of the year. Thus, there is a need to better understand the relationship between the detection of PRRSV in the wean-to-market category and its subsequent influence in the increased detection of PRRSV detection in adult/sow farms. However, limited research has been done to investigate this relationship.

To address the aforementioned research gap, we examine the relationship between PRRSV detection in the age categories wean-to-market vs. adult/sow farm and investigate the extent to which the wean-to-market PRRSV rate forecasts the adult/sow farm PRRSV rate. We first use statistical tools including correlation and regression to investigate to what extent the increase in PRRSV positive submissions in the wean-to-market is related to the PRRSV increase in adult/sow farms. The statistical analysis confirms that the increase in the PRRSV positive rate of wean-to-market precedes the increase in the adult/sow farms to a large extent. Then we establish a method based on the dynamic exponentially weighted moving average (EWMA) control chart [15] to predict PRRSV out-of-control points (i.e., signals) in adult/sow farms utilizing the PRRSV signals in wean-to-market. We also evaluate the accuracy of the prediction. We find out that the PRRSV signals in the wean-to-market category are largely informative in predicting the PRRSV signals in the adult/sow farms.

This research is original and creative in that, 1) it is the first to use statistical tools to investigate the relationship between PRRSV detection rates in two different age categories, and 2) it is also the first to leverage this relationship to forecast the signals in adult/sow farms. We expect that our findings will help the industry to justify and reinforce the implementation of bio-security and bio-contaminant practices to curb disease spread across farms.

## Materials and methods

**Fig 1:**
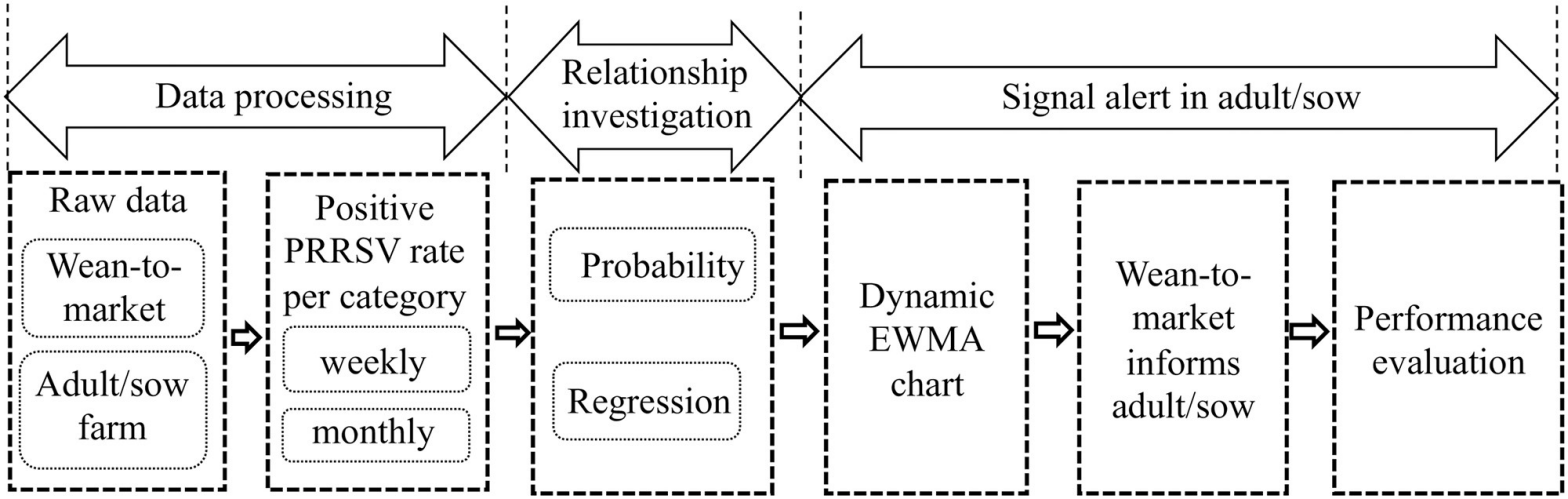
The schematic of the framework.

In this research, as shown in Fig 1, we first examined the characteristics shown in the monthly data and weekly data of the PRRSV positive rate for both wean-to-market and adult/sow farm age categories. Then we investigated the overall relationship between the PRRSV positive rates of two age categories by calculating conditional probabilities and conducting linear regression analyses. There was evidence showing that the increase in the PRRSV positive rate of wean-to-market precedes the increase in adult/sow farms. Thus, we created an alert model based on the dynamic EWMA control chart for PRRSV signals in adult/sow farms leveraging this relationship. We also quantified the accuracy of the alert model.

### Data source and management

Information for PRRSV testing over time was recovered from the SDRS project (www.fieldepi.org/SDRS). The time frame is from June 2007 to November 2019 which consists of 150 months or 660 weeks. Percentages of positive submissions (No. of positive submissions/total × 100) for the age categories adult/sow farm and wean-to-market were calculated by month and week. The total number of data points in the monthly and weekly PRRSV-detection-positive rates is 150 and 660, respectively. Submissions classified as adult/sow farms represent the samples collected and submitted for testing coming from replacement and breeding herds, whereas submission classified as wean-to-market represents the samples collected and submitted for testing coming from grower animals.

**Fig 2:**
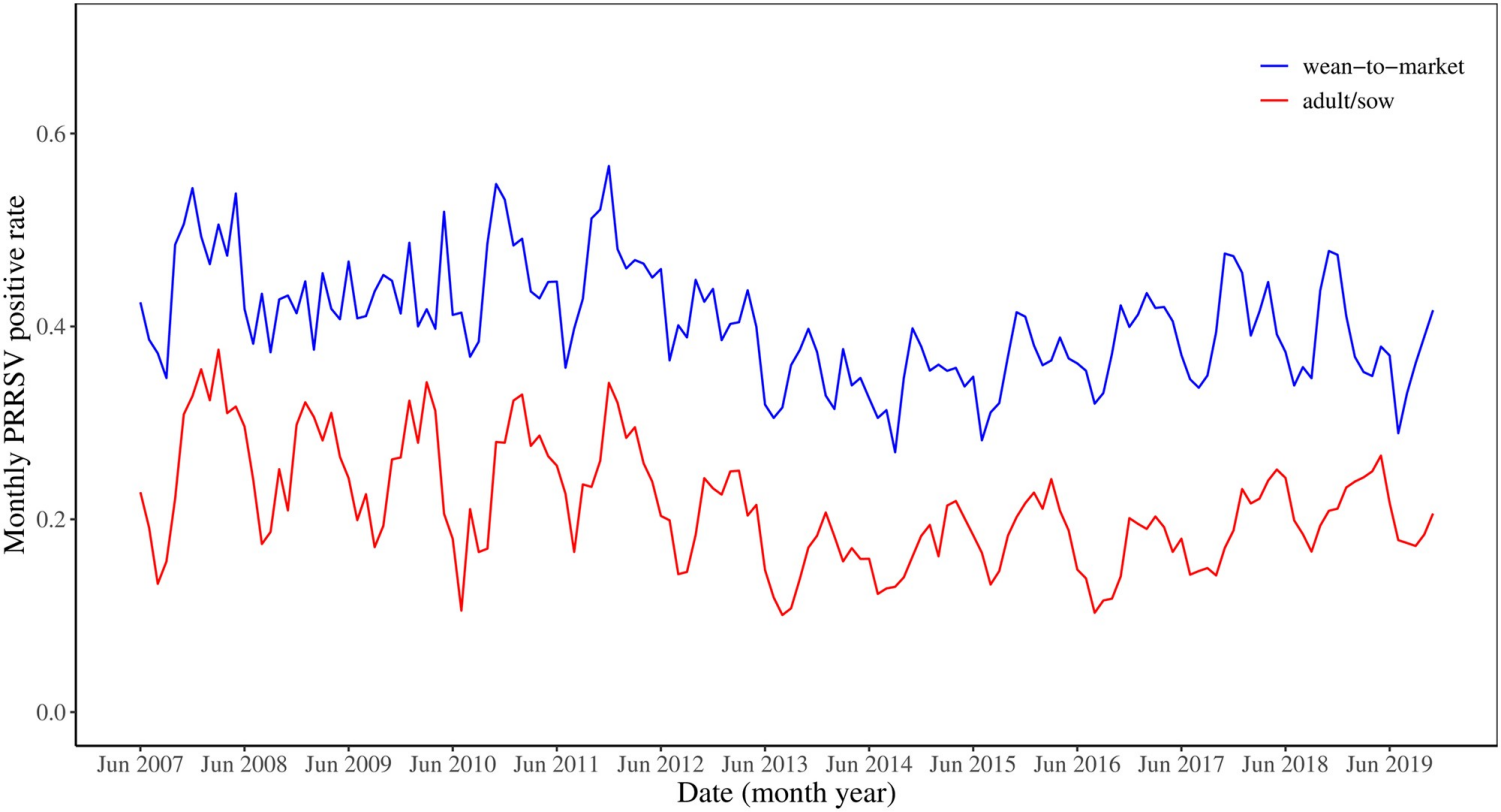
Monthly porcine reproductive and respiratory syndrome virus (PRRSV) positive rate time series by age categories from 2007 to 2019.

Fig 2 shows the monthly PRRSV positive rates between the first week of June 2007 (i.e., the 22nd week of 2007) to the first week of November 2019 of two age categories. The positive rates of wean-to-market are consistently much higher than that of adult/sow farms. There are apparent seasonal cyclic patterns in both of the PRRSV positive rates. The summer months have consistently smaller positive rates than the winter or spring months. The PRRSV positive rates reach the lowest level between the summer of 2013 and the fall of 2014. After this period, the positive rates start to increase gradually until the end of 2019. It seems the increased proportion of PRRSV positive rates of wean-to-market precedes that of the increase in adult/sow farms often, for example, in 2014-2016, and also in 2018 and 2019.

**Fig 3:**
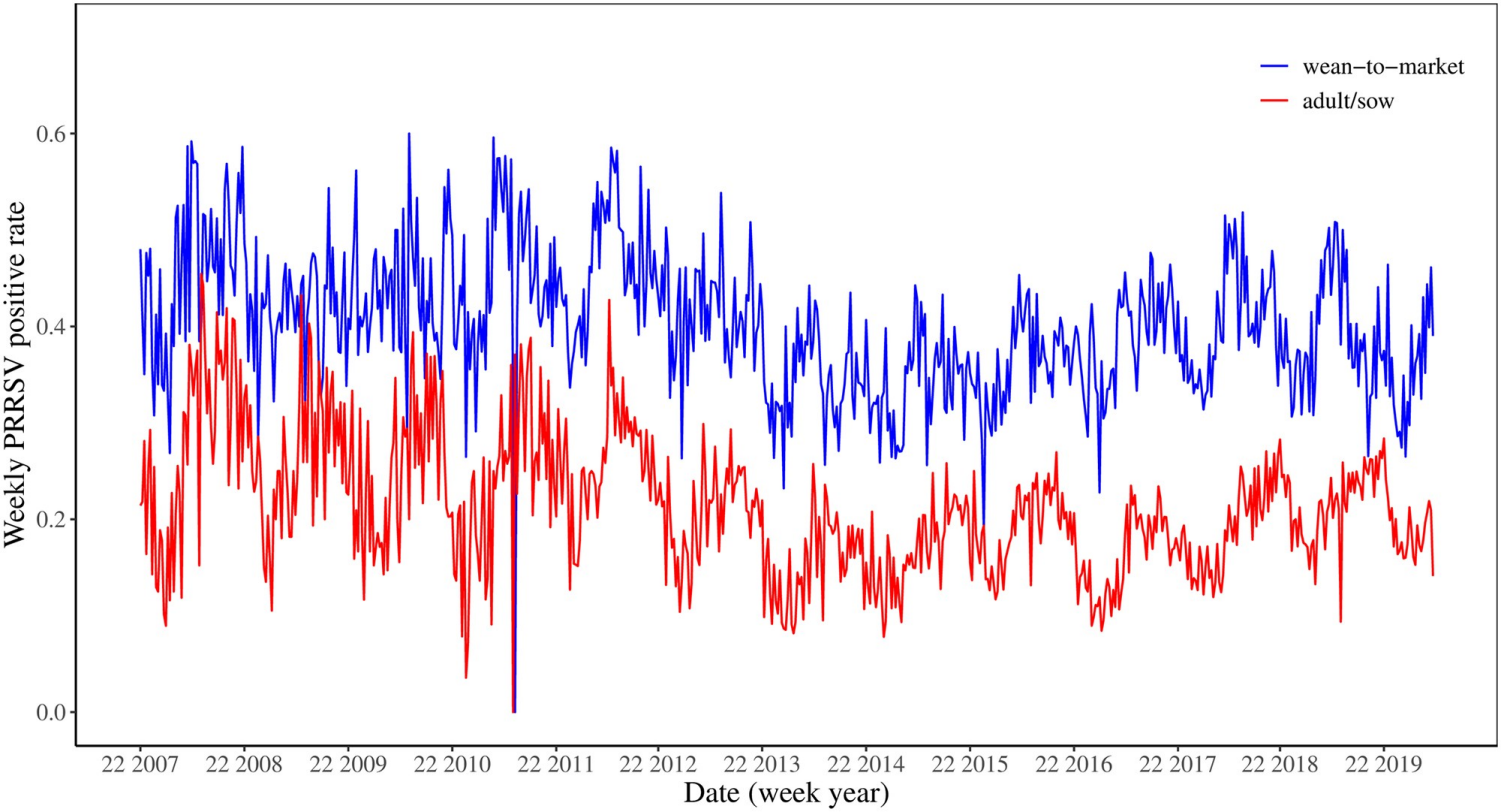
Weekly porcine reproductive and respiratory syndrome virus (PRRSV) positive rate time series by age categories from 2007 to 2019. The data begins on the 22nd week (i.e., the first week in June) in 2007.

Similar patterns are shown in the weekly PRRSV positive rates in Fig 3 where each year consists of 52 weeks.

### Relationship investigation

We examine the overall relationship between the PRRSV positive rates of two age categories by two methods: 1) calculating the conditional probabilities that adult/sow PRRSV increases given that wean-to-market PRRSV rate increases, and 2) comparing linear regression models with vs. without wean-to-market information for predicting adult/sow farm PPRSV positive rates.

#### Probability method

The initial investigation seeks to explore if the probability of PRRSV increases in the adult/sow given that wean-to-market PRRSV rate increases are significant and what is the potential lag between increases in the wean-to-market and adult/sow. We speculate that if the wean-to-market PRRSV rate increases from one time period to the next period, the likelihood that the adult/sow PRRSV rate will increase *m* time periods subsequent will be greater than the unconditional likelihood that the adult/sow PRRSV rate increases from one time period to the next.

In this article, we examined this relationship according to different lags by calculating the conditional probability *P*{*AS*(*t*) − *AS*(*t* − 1)>0|*WM*(*t* − *m*) − *WM*(*t* − *m* − 1)>0}, where *AS*(*t*) is the adult/sow farm PRRSV rate at time *t*, *WM*(*t*) is the PRRSV rate in the wean-to-market at time *t*, and *m* represents the number of time lags, *m* = 1, 2, 3, ⋯. The conditional probability that Event *A* occurs given that Event *B* has occurred denoted by *P*(*A*|*B*) reflects the impacts that the occurrence of *B* has on the probability that *A* occurs [16–18]. This conditional probability was compared to the unconditional probability that the adult/sow PRRSV rate increases or *P*{*AS*(*t*) − *AS*(*t* − 1)>0}. If the conditional probability is larger than the unconditional probability, it indicates that the increase of PRRSV in wean-to-market animals tends to precede the increase of PRRSV detection in adult/sow farms.

#### Linear regression with vs. without wean-to-market information

Linear regression is a powerful method when exploring the relationship between dependent variables and independent variables [19, 20]. Because our aim is to investigate the extent to which the wean-to-market PRRSV rate forecast the adult/sow PRRSV rate, a linear regression of the time series of the two age categories is expected to help determine if a relationship exists. However, the PRRSV rate data exhibits substantial variation over time. This variation makes it challenging to use the preceding period value to forecast the current value. Using a moving average time series model helps to eliminate some of the variability and capture the trend.

Denote the moving average *MA*_*AS*_(*t*) to be the moving average for the adult/sow farm for *N* time periods at time *t*, and *MA*_*WM*_(*t*) to be the moving average for the wean-to-market at time *t*. We used the moving average as an independent variable to forecast the actual value in the monthly series. We compared the forecast of the adult/sow PRRSV rate that used a model without the wean-to-market PRRSV rate (Model 1) to a model that used the wean-to-market PRRSV rate (Model 2) to help us assess the effect of knowing the wean-to-market PRRSV rate. The two models are as follows.

**Model 1**:
$$\begin{array}{c} AS\left(t+1\right)=\beta_{0}+\beta_{1}*MA_{AS}\left(t\right)+\epsilon , \end{array}$$ (1)
**Model 2**:
$$\begin{array}{c} AS\left(t+1\right)=\beta_{0}+\beta_{1}*MA_{AS}\left(t\right)+\beta_{2}*MA_{WM}\left(t\right)+\beta_{3}*MA_{WM}\left(t-1\right)+\epsilon , \end{array}$$ (2)

where *β*_0_, *β*_1_, *β*_2_, and *β*_3_ are regression parameters, and *ϵ* ∼ *N*(0, *σ*^2^) is the error term that is normally distributed with a mean of 0 and variance of *σ*^2^. Model 2 includes two variables representing the wean-to-market PRRSV rate: *MA*_*WM*_(*t*) is the moving average of wean-to-market rate one time period before the adult/sow rate and *MA*_*WM*_(*t* − 1) is the moving average of wean-to-market rate two time periods before the adult/sow rate. Since we expect that there is a lag between the wean-to-market positive rate and adult/sow positive rate, we included the two preceding time periods in the wean-to-market.

We chose a moving average of *N* = 3 months for each PRRSV rate. A 3-month moving average seems reasonable because it represents a season and reduces some of the monthly variations while still allowing for each month to individually contribute to the model.

The models were compared in multiple ways to see the effect of including the wean-to-market data in forecasting the adult/sow prevalence. Specifically, the coefficient of determination *R*^2^ and the p-values of regression coefficients reveal the relationship in different aspects [21] and were compared. First, *R*^2^ values of the two models were compared to understand how much greater *R*^2^ in Model 2 is than in Model 1. Second, the p-values of *β*_2_ and *β*_3_ were calculated to test the significance of including the moving average of the wean-to-market data.

### Signal alert by the dynamic EWMA chart

The results of the Relationship Investigation Section (detailed in the Results Section) show evidence that increases in the PRRSV positive rate of the wean-to-market phase precede increases in the PRRSV positive rate of adult/sow farm to a certain extent, which leads to the hypothesis that the increased PRRSV activity in the wean-to-market category leads to virus transmission in the adult/sow farms. Thus, we used the signals detected in the wean-to-market category to alert the signals in the adult/sow farms. Usually, if viruses transfer from one farm to another and result in PRRSV signals, it takes less than two months. Therefore, we only considered the PRRSV signals in adult/sow farms within two months of the signals in wean-to-market as the consequence of virus transfer from wean-to-market farms.

We used control charts to identify points out of control (i.e., signals) to capture signals or large increases in the PRRSV rate in these two time series. Then we examined if a signal in the wean-to-market category leads to a signal in the adult/sow farms. Control charts are commonly used tools in statistical process control to monitor a process and detect the significant variation [22, 23]. De Vries et al. [24] demonstrate that carefully constructed control charts are powerful methods to monitor animal production systems, and are also useful to trace shifts and deviations in commercial swine farming.

The EWMA chart [15] is a type of the most commonly used control charts for detecting small shifts which are less than 1.5 times of sample variance with respect to the process mean [25]. The EWMA chart monitors the exponentially-weighted moving average of all prior sample means so that the recent samples are weighted more than the distant samples [26]. Krietera, et al. [27] applied the EWMA chart to detect the process change in pig production using the sow/farm datasets and yielded great detection performance. In summary, the EWMA chart is powerful in process variation detection and can be applied in our datasets. Therefore, in this article, we captured signals in wean-to-market using a EWMA control chart to create alerts for signals in the adult/sow farms. We constructed two EWMA control charts, one chart based on the weekly PRRSV rate for wean-to-market and the other chart based on the weekly PRRSV rate for adult/sow farms.

The standard EWMA control chart assumes the mean of the data does not change. However, we see that the mean of the PRRSV rate changes for both data sets around Summer 2013 as shown in Figs 2 and 3, which coincides with the increased bio-security practices in US swine herds [28] in response to the emergence of porcine epidemic diarrhea virus in the spring of 2013 [29]. Thus, we created the dynamic EWMA chart by sliding a moving window with a size of 52 weeks to create dynamic mean and limits since 52 weeks represent the annual period in the data.

The EWMA control chart smooths data by adding a smoothing parameter λ to a moving average model and a smaller value of λ implies stronger smoothing [30]. The monitored statistic at time *t* denoted by *z*_*t*_ is calculated by *z*_*t*_ = λ*x*_*t*_ + (1 − λ)*z*_*t*−1_, where *x*_*t*_ is the original time series data [31]. The control limits for a process with *n* samples are defined as $T\pm L\frac{S}{\sqrt{n}}\sqrt{\frac{\lambda }{2-\lambda }\left[1-{\left(1-\lambda \right)}^{2t}\right]}$, where *T* and *S* are the estimates of the long-term process mean and standard deviation of historical data,respectively. *L* is a multiple of the standard deviation that establishes the control limits. *L* is typically set at 3, and smaller *L* can be used for small values of λ. The data values greater than the upper control limit indicate signals.

We chose λ = 0.8 and *L* = 1.2 for the dynamic EWMA control charts of the PPRSV positive rates in wean-to-market and adult/sow farms. Different recommendations exist for λ, such as 0.75 ≤ λ ≤ 0.95 and 0.7 ≤ λ ≤ 0.8 [15, 30]. Montgomery [15] suggests λ could be reduced for smaller values of *L*. Our goal in constructing these control charts is to identify enough points that exceed the upper control limit to understand the relationship between large PRRSV rates in the wean-to-market and adult/sow data. However, if the upper control limit is too small, too many points will exceed the upper control limit. We experimented with different values of *L* and found that *L* = 1.2 provides a reasonable number of signals in the EWMA control chart.

To take advantage of the signals in the wean-to-market category to alert the signals in the adult/sow farms, we assume if a wean-to-market signal occurs, then we should be seeing an adult/sow farm signal. Denote the signals in adult/sow farms and wean-to-market category as signal_*AS*_(*t*) and signal_*WM*_(*t*), respectively. For the adult/sow farms, we define an event signal_*AS*_(*t* + 1 : *t* + *m*) as the occurrence of at least one data point in the adult/sow control chart exceeding the upper control limit from time periods *t* + 1 to *t* + *m*, where *m* is a positive integer. Similarly, we define the event signal_*WM*_(*t*) as the occurrence of the observation at time *t* in the wean-to-market category exceeding its upper control limit.

The number of signals in these two control charts are used to calculate the probabilities *P*{signal_*AS*_(*t* + 1 : *t* + *m*)|signal_*WM*_(*t*)} and *P*{signal_*WM*_(*t* − 1 : *t* − *m*)|signal_*AS*_(*t*)} to evaluate the performance of the alert effectiveness. The probability *P*{signal_*AS*_(*t* + 1 : *t* + *m*)|signal_*WM*_(*t*)} is the conditional probability that a signal occurs in the adult/sow control chart within *m* units of time given that a signal occurs in the wean-to-market control chart at time *t*, which represents the probability of a correct forecast or alarm. Correspondingly, 1 − *P*{signal_*AS*_(*t* + 1 : *t* + *m*)|signal_*WM*_(*t*)} is the false alarm probability. Thus, larger *P*{signal_*AS*_(*t* + 1 : *t* + *m*)|signal_*WM*_(*t*)} results in a lower false alarm rate. Namely, once a signal in the wean-to-market farms occurs, we have higher confidence that a signal will occur in the adult/sow farms. On the other hand, the probability *P*{signal_*WM*_(*t* − 1 : *t* − *m*)|signal_*AS*_(*t*)} is the conditional probability that a signal occurs in the wean-to-market control chart at least *m* units of time prior given that a signal occurs in the adult/sow control chart, which represents the percentage of signals in the adult/sow farms that are preceded by a signal in the wean-to-market farms. If *P*{signal_*WM*_(*t* − 1 : *t* − *m*)|signal_*AS*_(*t*)} is large, then we are confident that most signals in the adult/sow farms could be predicted by signals in the wean-to-market category.

As we explained before, if the PRRSV transfers from one farm to another farm and leads to signals, it usually takes less than two months. Thus, we only considered the signals of PRRSV in adult/sow farms within two months of the signals in wean-to-market as the consequence of virus transfer from wean-to-market category and set *m* = 8. When we counted the number of signals in the dynamic EWMA control charts, we combined signals within 4 weeks in both series to avoid repetitive counts of signals.

## Results and discussion

In this section, we first present the evidence of the preceding effect of PRRSV detection in wean-to-market over adult/sow farms from two perspectives. Then we show the results of signal detection for PRRSV signals in adult/sow farms informed by the detection of PRRSV signals in wean-to-market category using the dynamic EWMA control charts.

### Evidence of preceding effect

#### Probability methods

The data set includes 150 months for both the wean-to-market and the adult/sow PRRSV rates. With 150 months of rates, there are 149 differences between consecutive months. For the adult/sow farms, there are 79 increases out of these 149 differences or there is a 53% chance that the adult/sow PRRSV rate increases from one month to the next month.

The conditional probability that the adult/sow PRRSV rate increases given that the wean-to-market PRRSV rate increases *m* months prior is given in Table 1. These results suggest the adult/sow PRRSV rate and the wean-to-market PRRSV rate 1 and 2 months prior exhibit some probabilistic dependence. The conditional probabilities are 60% and 61% for 1 and 2 months, respectively, which are a little greater than the unconditional probability of 53%. After 2 months, the conditional probability declines closer to the unconditional probability.

**Table 1:** The conditional probability that the adult/sow PRRSV rate increases given that the wean-to-market PRRSV rate increases *m* months prior: *P*{*AS*(*t*) − *AS*(*t* − 1) > 0|*WM*(*t* − *m*) − *WM*(*t* − *m* − 1) > 0}.

Month lag *m*	1	2	3	4	5
Probability	0.603	0.611	0.521	0.443	0.571

#### Linear regression with and without wean-to-market

Since there seems to be some dependence between the adult/sow and the wean-to-market PRRSV rates, we constructed the linear regression model to include the moving average of the wean-to-market 1 and 2 months prior to the adult/sow farms. Table 2 displays the results of Models 1 and 2 including the parameter estimates, the 95% confidence interval, the *R*^2^, F-statistic, and the p-value. Notice that Model 1 in Eq 1 uses the moving average of the adult/sow PRRSV rate to predict the adult/sow PRRSV rate one month later while Model 2 in Eq 2 uses the moving average of the wean-to-market 1 and 2 months prior to the adult/sow PRRSV rate in addition to the moving average of the adult/sow 1 month prior.

**Table 2:** Results of Models 1-2 with the statistics of model parameters and performance.

Model	Parameter	Estimate	95% Confidence interval	*R*^2^	F-statistic	p-value
Model 1	*β*_0_	0.017	[0.027, 0.084]	0.469	127.4	10^−21^
	*β*_1_	0.922	[0.613, 0.873]			
Model 2	*β*_0_	−0.033	[−0.087, 0.021]	0.636	82.5	10^−31^
	*β*_1_	0.561	[0.381, 0.741]			
	*β*_2_	1.083	[0.811, 1.356]			
	*β*_3_	−0.767	[−1.070, −0.464]			

*R*^2^ is the coefficient of determination.

When comparing Models 1 and 2, we observe that Model 2 with the wean-to-market item performs better than Model 1, which is expected because Model 2 includes more independent variables. Including the moving average of the wean-to-market increases the *R*^2^ value from 0.47 to 0.64. For Model 2, the regression parameters for the moving average of the wean-to-market 1 and 2 months prior are both significant at the 5% level as their 95% confidence intervals do not include 0. The moving average of the wean-to-market 1 month prior has a positive effect (*β*_2_ > 0), and the moving average of the wean-to-market 2 months prior has a negative effect (*β*_3_ < 0). Both parameters have a larger magnitude than the parameter associated with the moving average of the adult/sow PRRSV rate one month earlier (*β*_1_).

**Fig 4:**
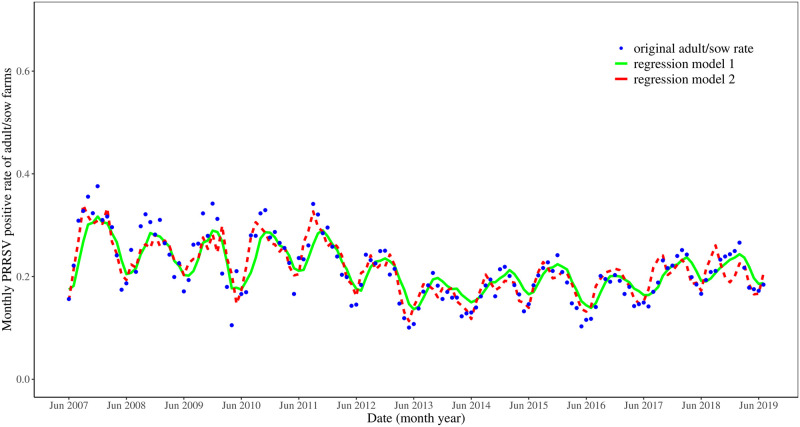
Regression models for the porcine reproductive and respiratory syndrome virus (PRRSV) positive rates in adult/sow farms.

Fig 4 displays the adult/sow farm original data and the predicted values for regression Models 1 and 2. The two models have similar predictions. Model 2 seems to forecast the really large PRRSV rates better than Model 1 especially in the first half of the data set. In general, the signals in Model 2 precede the signals in Model 1.

### Alerts for PRRSV signals in adult/sow farms

**Fig 5:**
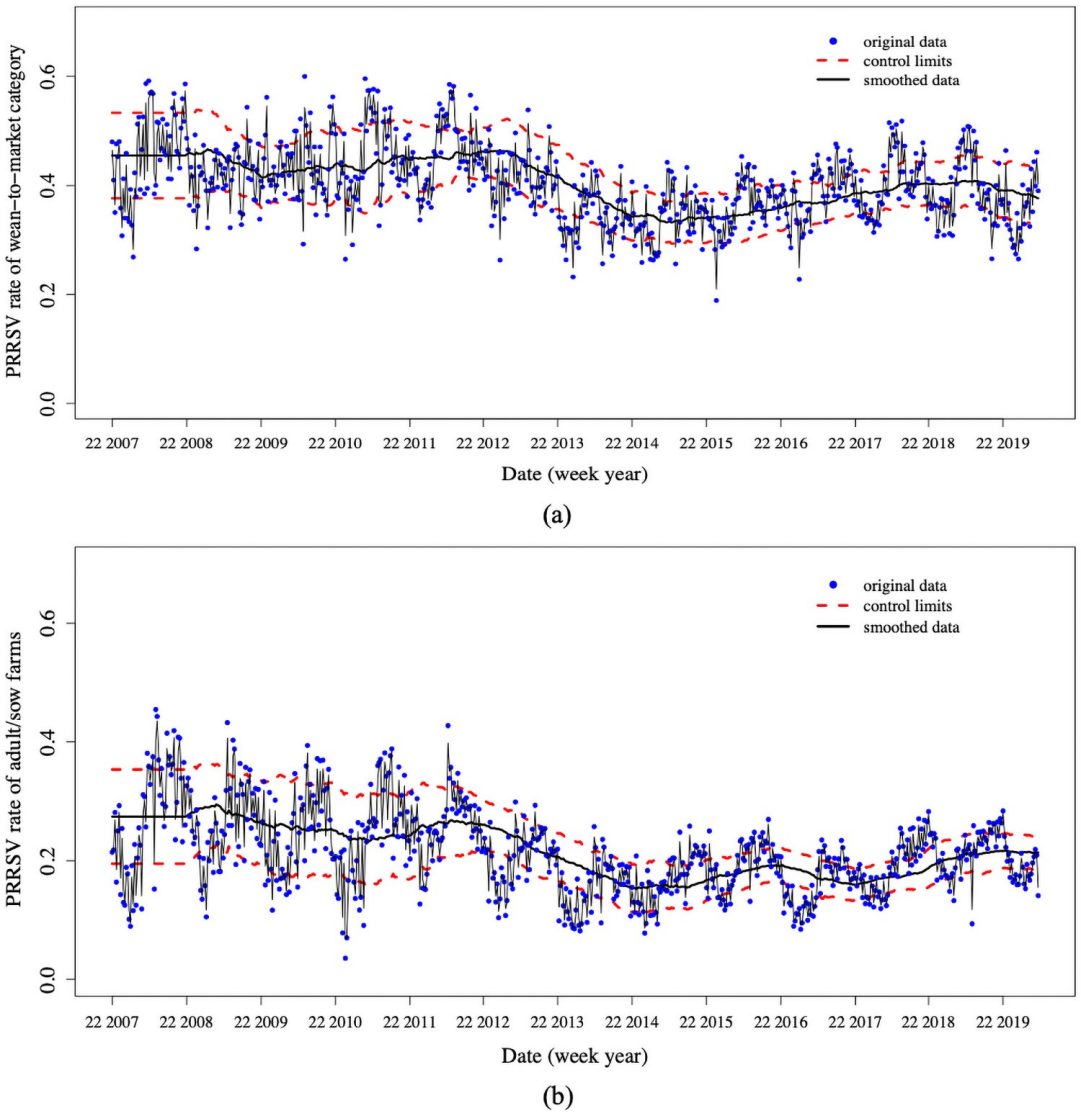
The dynamic EWMA control charts with a moving window having the size of 52 weeks. (a) The chart for detecting the PRRSV signals in wean-to-market categories. (b) The chart for detecting the PRRSV signals in in adult/sow farm.

Both the probability methods and the regression show that the proportion of positive submissions in wean-to-market precedes the increase in adult/sow farms. This suggests that signals in wean-to-market could inform future signals in adult/sow farms. Fig 5 shows the dynamic EWMA control charts with both the original data and the EWMA model for the PRRSV positive rates in wean-to-market and adult/sow farms based on the weekly data.

In summary, there are 124 signals in the adult/sow farms, and 115 signals in the wean-to-market category. Among all the wean-to-market signals, there are 97 signals that are followed by a signal in the adult/sow farms within the next two months. That is, the probability *P*{signal_*AS*_(*t* + 1 : *t* + 8)|signal_*WM*_(*t*)} = 97/124 = 0.721, indicating that if a signal occurs in the wean-to-market control chart at time *t*, there is a 72.1% probability that a signal will occur in the adult/sow farms within 8 weeks, and the false alarm rate is 27.9%. On the other hand, there are 83 signals in the adult/sow farms that have a signal in the wean-to-market within two months prior. That is, the probability *P*{signal_*WM*_(*t* − 1 : *t* − 8)|signal_*AS*_(*t*)} = 83/115 = 0.782, indicating that 78.2% of the signals in the adult/sow farms were preceded by at least one signal in wean-to-market within 8 weeks, thus could be identified in advance. There are 21.8% of signals in the adult/sow farms that are hard to predict with the wean-to-market signals.

Overall, we find that the signals in the wean-to-market PRRSV rate series inform the PRRSV signals in adult/sow farms in the next two months with an accuracy of 0.78 while the false alarm rate is approximately 0.28. The result is promising since most of the signals in adult/sow farms are able to be predicted based on the wean-to-market PRRSV rate.

### Discussion

This research is the first to use statistical tools to investigate to what extent there is a connection/relationship between PRRSV detection rates in two different age categories (wean-to-market and adult/sow farms). It is also the first to leverage this relationship to inform the signals in adult/sow farms. Three methods of analysis were applied to model the relationship between the PRRSV rate in the wean-to-market and the adult/sow farms. Each analysis demonstrates that the PRRSV rate in the wean-to-market category seems to have certain predictive value in the adult/sow farm PRRSV rate 1-2 months later.

The probability that the adult/sow farm PRRSV rate increases from one month to the next month is 53%. If this probability that the PRRSV rate in adult/sow farms increases is conditioned on an increase in the monthly wean-to-market PRRSV rate two months prior, the chance of an increase in adult/sow farms is 61%. This suggests some probabilistic dependence between increases in the two rates, though the dependence is not too strong.

The regression model using moving averages to predict the adult/sow farm PRRSV rate also suggests that the wean-to-market PRRSV rate can forecast the adult/sow farm PRRSV rate by approximately 1-2 months. The coefficients corresponding to the moving averages of the wean-to-market PRRSV rate are statistically significant at the 5% level, and the magnitude of each of those coefficients exceeds the magnitude of the coefficient corresponding to the adult/sow farm moving average one month prior to the actual adult/sow farm PRRSV rate. The *R*^2^ in Model 2 is 0.64 which is 0.17 larger than *R*^2^ for Model 1 (without the wean-to-market item). Since *R*^2^ indicates the proportion of variation in the dependent variable explained by the model, *R*^2^ = 0.64 indicates that a lot of variation in the adult/sow farm PRRSV rate is not explained by the moving averages. Clearly, many other factors also contribute to the explanation of the adult/sow farm PRRSV rate.

The dynamic EWMA model explicitly models signals or spikes in the PRRSV rate and suggests that high PRRSV rates in the wean-to-market category of a week can indicate that high PRRSV rates are likely to occur in the adult/sow farms within the next two months. If a signal occurs in the wean-to-market category, we assessed that there is a 72% chance that a signal will occur in the adult/sow farms within the next 8 weeks. Based on the data from 2007 to 2019, we calculated that there is a 48% chance that any randomly selected 8-week period in the adult/sow farms will exhibit a signal or point that exceeds the EWMA upper control limit. Thus, the signals in the wean-to-market category indicate a fairly sizable increase in our confidence that the adult/sow farms will experience a spike in PRRSV rates. These findings provide useful information to help the swine industry to be well-prepared for the upcoming challenges.

In addition to the insights generated by this research, this work is also important because it demonstrates the types of statistical analysis and models that veterinary practitioners could undertake in order to assess potential relationships among animal herds or farms in different stages of development, especially as it relates to disease rates. Understanding and modeling the effect that one type of animal herd may have over another type of animal herd can lead to better strategies to prevent the spread of viruses and diseases.

Despite the usefulness of these findings in providing a greater understanding of the relationship between the PRRSV rates in wean-to-market and adult/sow farms, we recognize this research has several limitations. The statistical analyses of these models are relatively simple, and perhaps more complicated analysis and models could identify a stronger relationship or even less of a relationship. Future work could investigate the importance of other factors such as temperature and humidity in predicting PPRSV signals in adult/sow farms. As Figs 2 and 3 show, the PRRSV rates in both wean-to-market and adult/sow farms exhibit a strong seasonal or cyclic pattern. Our models could be unknowingly reflecting these seasonal effects. Models that account for seasonal effects may demonstrate less of a relationship between wean-to-market and adult/sow PRRSV rates. Finally, the PRRSV rate exhibits a sharp decrease in 2013, and it may be more important to analyze only the data after 2013 if we are interested in understanding the present or future relationship.

Notice that there is no similar research to our best knowledge. The only closely relevant work by Trevisan, et al. [14] found that the detected outbreak signals in adult/sow farms were preceded by an increased detection of PRRSV in the wean-to-market category. However, the insights about the relationship of PRRSV outbreak signals between adult/sow and wean-to-market age categories in Trevisan, et al. [14] were solely based on exploratory graphs, which lacks solid statistical support. This research investigates the relationship with probability methods and concrete statistical models, which reveals the preceding effects of PRRSV detection in wean-to-market over adult/sow farms with sound mathematical justification.

## Conclusions

In this research, to investigate to what extent the wean-to-market PRRSV rates help to predict the signals in the adult/sow farm PRRSV positive rate, we first analyzed the monthly PRRSV positive rates by conditional probabilities and regression. The results indicate that there exists a precedence relationship between the PRRSV positive rates of wean-to-market and adult/sow farms. Then we leveraged the signals in the weekly wean-to-market data to inform the signals in the adult/sow farms based on the dynamic EWMA charts. With the detection of a signal in the wean-to-market PRRSV rate series, there is a large probability that there would be PRRSV signals in adult-sow farms within the next two months.

Using statistical models, we demonstrated the preceding effect and contributions of the wean-to-market PRRSV detection rate on the macro-epidemiological levels of PRRSV detection rate in the adult/sow farms. The developed models can be consistently applied to the SDRS data and predict changes in the expected adult/sow farm PRRSV detection rate 2 months in advance, thus informing stakeholders of relevant and potential changes in PRRSV detection patterns. Producers and veterinarians can use such information to reinforce bio-security and bio-contaminant practices in preparedness efforts to reduce disease spread across farms. Additionally, this study demonstrated that PRRSV detection in wean-to-market is an important component in the macro-epidemiological aspects of PRRSV detection. This, in sum, raised the awareness that further development and improvements in the strategies to control PRRSV in the wean-to-market age category is expected to have an additional benefit to adult/sow farms.
